# A Sustained Immune Response Supports Long-Term Antiviral Immune Priming in the Pacific Oyster, Crassostrea gigas

**DOI:** 10.1128/mBio.02777-19

**Published:** 2020-03-10

**Authors:** Maxime Lafont, Agnès Vergnes, Jeremie Vidal-Dupiol, Julien de Lorgeril, Yannick Gueguen, Philippe Haffner, Bruno Petton, Cristian Chaparro, Celia Barrachina, Delphine Destoumieux-Garzon, Guillaume Mitta, Benjamin Gourbal, Caroline Montagnani

**Affiliations:** a IHPE, University of Montpellier, CNRS, Ifremer, University of Perpignan Via Domitia, Montpellier, France; b IHPE, University of Montpellier, CNRS, Ifremer, University of Perpignan Via Domitia, Perpignan, France; c Ifremer, LEMAR UMR6539, Argenton-en-Landunvez, France; d MGX, Biocampus Montpellier, CNRS, INSERM, University of Montpellier, Montpellier, France; French National Centre for Scientific Research; CIML

**Keywords:** innate immunity, priming, OsHV-1, antiviral response, immune memory, oyster, POMS, poly(I·C), interferon, transcriptomic

## Abstract

Over the last decade, innate immune priming has been evidenced in many invertebrate phyla. If mechanistic models have been proposed, molecular studies aiming to substantiate these models have remained scarce. We reveal here the transcriptional signature associated with immune priming in the oyster Crassostrea gigas. Oysters were fully protected against Ostreid herpesvirus 1 (OsHV-1), a major oyster pathogen, after priming with poly(I·C), which mimics viral double-stranded RNA. Global analysis through RNA sequencing of oyster and viral genes after immune priming and viral infection revealed that poly(I·C) induces a strong antiviral response that impairs OsHV-1 replication. Protection is based on a sustained upregulation of immune genes, notably genes involved in the interferon pathway and apoptosis, which control subsequent viral infection. This persistent antiviral alert state remains active over 4 months and supports antiviral protection in the long term. This acquired resistance mechanism reinforces the molecular foundations of the sustained response model of immune priming. It further opens the way to applications (pseudovaccination) to cope with a recurrent disease that causes dramatic economic losses in the shellfish farming industry worldwide.

## Abstract (precis)

In the last decade, important discoveries have shown that resistance to reinfection can be achieved without a functional adaptive immune system, introducing the concept of innate immune memory in invertebrates. However, this field has been constrained by the limited number of molecular mechanisms evidenced to support these phenomena. Taking advantage of an invertebrate species, the Pacific oyster (Crassostrea gigas), in which we evidenced one of the longest and most effective periods of protection against viral infection observed in an invertebrate, we provide the first comprehensive transcriptomic analysis of antiviral innate immune priming. We show that priming with poly(I·C) induced a massive upregulation of immune-related genes, which control subsequent viral infection, and it was maintained for over 4 months after priming. This acquired resistant mechanism reinforces the molecular foundations of the sustained response model of immune priming. It opens the way to pseudovaccination to prevent the recurrent diseases that currently afflict economically or ecologically important invertebrates.

## INTRODUCTION

Since the last decade, a large body of evidence has been accumulated demonstrating that innate immune systems can confer increased protection to reinfection. Evidence in plants and invertebrates lacking classical adaptive immunity indicates that these organisms can develop a stronger response to a challenge if they have been previously stimulated with the same or with another agent. Since this phenomenon cannot be associated with the conventional adaptive immunity found in vertebrates, it has been associated with the term innate immune priming, trained immunity, or innate immune memory (1, 2).

However, regardless of the accumulating phenotypic evidence of immune priming, understanding of its mechanistic foundations is currently very scarce. Several mechanistic models have been proposed to explain the implementation of immune priming: (i) a biphasic response with a first response to priming followed by an extinction phase and either a similar but faster and stronger secondary response to infection (named recall response) or a qualitatively distinct secondary response (named immune shift) and (ii) a unique response activated after priming, not followed by an extinction phase, and maintained through the secondary infection (named sustained response) (3). But so far, only a few molecular mechanisms have been evidenced regarding innate immune priming. Epigenetic reprogramming has been considered the main mechanism driving this phenomenon in mammalian innate immune cells or in plants, allowing short-term adaptations of the epigenome (4, 5). In addition, reports have suggested the implication of certain classes of multigene family receptors (Down syndrome cell adhesion molecules [DSCAMS] and fibrinogen-related proteins [FREPs]) but also immune effectors (antimicrobial peptides) and cellular processes (phagocytosis and hematopoietic proliferation) in immune priming (6–11). In spite of these scattered data, a key question still pending is that of the molecular foundations supporting these mechanistic models (12).

Evidence of antiviral innate immune priming has been obtained in the Pacific oyster, Crassostrea gigas (or Magallana gigas, Thunberg 1793), a main aquaculture species in the world that belongs to Lophotrochozoa, one of the widest groups in the animal kingdom that is still poorly explored regarding these processes. In this species, the herpes-like virus Ostreid herpesvirus 1 (OsHV-1) microvariant (μvar) triggers Pacific oyster mortality syndrome (POMS), which has plagued the oyster production worldwide for more than a decade, with no existing prophylactic or therapeutic treatments (13, 14). Recently, we showed that a noninfectious elicitor, the viral mimic synthetic double-stranded RNA (dsRNA) called poly(I·C), could induce protection of the oyster against that virus (15). Long-term protection (up to 5 months) was observed either in experimental infections or in the environment during a disease outbreak (16). However, we still lack a comprehensive view of the molecular basis involved in this antiviral immune priming. This requires the implementation of transcriptomic approaches to monitor global changes in host gene expression upon immune priming and subsequent immune challenge. To the best of our knowledge, such an approach has been implemented in only a few models. In the gastropod Biomphalaria glabrata (freshwater snail), challenged by the parasite Schistosoma mansoni (17, 18), and in the red flour beetle (19), data revealed an important immune shift conferring protection against a secondary infestation.

Given the exceptional long-term and efficiency of the antiviral protection demonstrated in the oyster, we believe that it represents a useful model system in which the evolution and mechanistic basis of innate immune priming can be studied. We used an RNA sequencing (RNA-seq) approach to study the temporal dynamics of antiviral immune priming in C. gigas. We investigated the response to poly(I·C) in highly virus-susceptible oysters and showed that the transcriptional dynamics of immune priming is characterized by a sustain transcriptomic remodeling of the oyster response and the induction of a potent immune response conferring long-term protection against viral infection. These results offer an attractive approach for enhancing immune capacities and open the way to pseudovaccination applications to fight pathogenic viruses causing high mortalities in the shellfish farming industry.

## RESULTS

### Poly(I·C) priming induces resistance to OsHV-1 infection in highly susceptible oysters.

**FIG 1:**
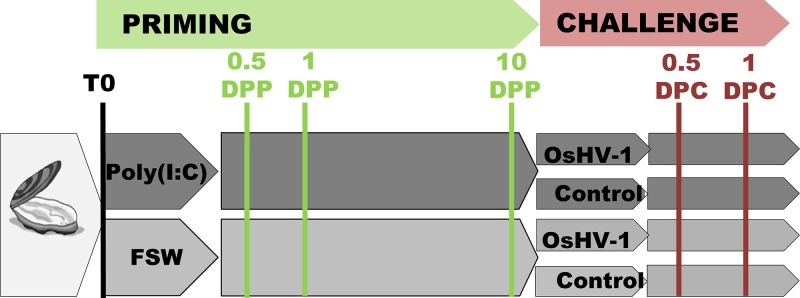
Experimental design used to identify molecular basis underlying poly(I·C) priming. Specific-pathogen-free (SPF) oysters, highly susceptible to Pacific oyster mortality syndrome, were anesthetized before being primed with poly(I·C) or filtered seawater (FSW). Ten days postpriming (10 DPP), oysters from each condition were challenged with OsHV-1 inoculum (1.32 × 10^8^ copies of DP gene per oyster) or OsHV-1-free inoculum (control). A part of the initial batch of oysters was kept untreated during the experiment. Survival of oysters was monitored for 10 days postchallenge. Three pools of 15 oysters for each condition were sampled postpriming (T0, 0.5 day, 1 day, and 10 days) and postchallenge (DPC) (0.5 day and 1 day) for viral load analyses or RNA sequencing.

**FIG 2:**
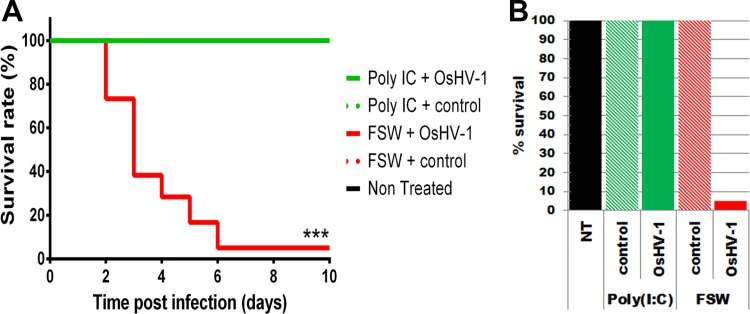
Poly(I·C) priming induces a phenotypical reversal upon OsHV-1 challenge. (A) Kaplan-Meier survival curves of oysters were primed by injection with poly(I·C) (19 μg g^−1^ of oyster) or filtered seawater as a control or left nontreated before being challenged by injection, 10 days postpriming, with an OsHV-1 inoculum (1.32 × 10^8^ copies of DP gene per oyster; solid lines) or with a control inoculum (OsHV-1-free, dotted lines) or left nontreated. All conditions except FSW plus OsHV-1 demonstrated 100% survival rates and appear hidden and merged with other ones reaching the same survival rate. Survival in each group of 60 oysters was monitored for 10 days after challenge. ***, *P* value < 0.0001, log rank test; *n* = 60. (B) Graph representing the final survival rates (10 days postchallenge) of oyster batches not treated (NT) or injected with poly(I·C) or FSW.

To characterize the molecular foundations underlying innate immune priming in the oyster, we used a biparental oyster family (family no. 11 [F_11_]) highly susceptible to Pacific oyster juvenile oyster syndrome (POMS) (14). This family reached 100% mortality when exposed to POMS. In the present study, F_11_ oysters were primed with poly(I·C) and experimentally challenged with OsHV-1 10 days postpriming (DPP). We monitored survival rates and virus loads and performed RNA-seq on postpriming and postchallenge samples (Fig. 1). Oysters injected with a noninfectious inoculum and nontreated oysters showed 100% survival at the end of the experiment (Fig. 2). Oysters injected with sterile (filtered) seawater (FSW) and challenged with OsHV-1 (priming controls) were highly susceptible to OsHV-1, with only 5% survival at the end of the experiment (10 days postchallenge [DPC]). Remarkably, poly(I·C) priming led to 100% protection after OsHV-1 challenge, with a significant difference of survival rate compared to that of the controls.

**FIG 3:**
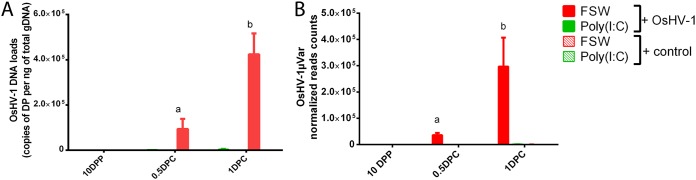
Poly(I·C) stimulation restrains OsHV-1 replication in the oyster. The OsHV-1 DNA load was quantified by quantitative PCR and expressed as viral genomic units per nanogram of total oyster DNA (A); viral replication was estimated by the total number of RNA-seq reads mapped on the OsHV-1μVarA genome (B). Virus dynamics was followed at different times after pathogenic (OsHV-1 inoculum) or nonpathogenic (control inoculum) challenges. Challenges were realized 10 days after priming with FSW or poly(I·C). Different letters above bars indicate statistically significant differences for each time compared to 10 DPP using one-way ANOVA (a, *P* value < 0.05; b and c, *P* value < 0.001; *n* = 3 pools of 15 oysters).

Dynamic of viral infection was monitored (i) by quantifying OsHV-1 genomic DNA (gDNA) by quantitative PCR (qPCR) in whole oyster tissues and (ii) by estimating the number of viral transcripts after challenge using an RNAseq approach (Fig. 3; see also Table S1 in the supplemental material). Viral DNA loads were 200 times lower in oysters primed with poly(I·C) and challenged with OsHV-1 at 1 DPC (Fig. 3A). Viral loads could not be compared at day 10 due to high mortality rates in priming controls. In the latter, virus replication was active from 0.5 DPC and increased at 1 DPC, with 8 times more transcripts (Fig. 3B). For oysters primed with poly(I·C), only 8 reads (on average) could be detected at 0.5 DPC and there was no significant increase at 1 DPC. This represented about 4,600 and 700 times fewer transcripts than under control conditions at 0.5 and 1 DPC, respectively. No mortalities, viral DNA, or transcripts were detected in negative controls (i.e., oysters challenged with noninfectious inoculum or untreated oysters).

10.1128/mBio.02777-19.6 TABLE S1 Library and mapped reads on the *C. gigas* genome or OsHV-1 genome used for RNA-seq analyses. Download Table S1, XLSX file, 0.01 MB. Copyright © 2020 Lafont et al. 2020 Lafont et al. This content is distributed under the terms of the Creative Commons Attribution 4.0 International license.

Together, these results show that poly(I·C) priming and FSW injection led to extremely contrasted phenotypes upon OsHV-1 challenge. In nonprimed oysters, high replication of OsHV-1 led to high viral loads, associated with low oyster survival rates. In poly(I·C)-primed oysters, virus replication was efficiently impaired, with low viral loads and high survival rates. Priming with poly(I·C) thus modified the phenotype of oysters in response to OsHV-1 infection from highly susceptible to resistant.

### Poly(I·C) priming induces a strong innate immune response.

**FIG 4:**
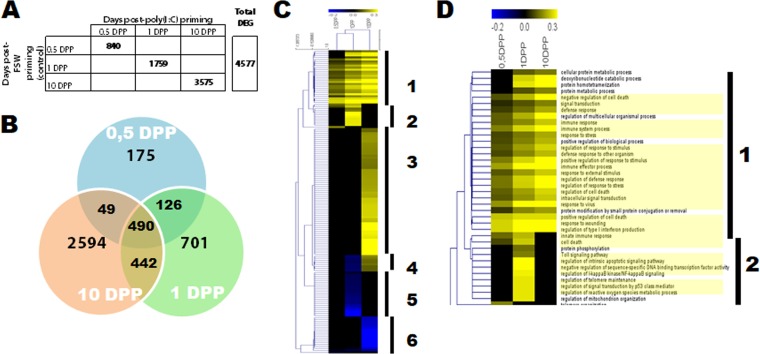
Poly(I·C) stimulates a strong innate immune response in oysters. (A) Comparisons of transcriptomes were performed between poly (I·C) and FSW-treated oysters at 0.5, 1, and 10 DPP using the DESeq2 package (FDR < 0.05). The table indicates the number of differentially expressed genes (DEG) between conditions and the total number of nonredundant DEG across all conditions (4,577). (B) Venn diagram representing the 4,577 DEG in each comparison. (C) Heat map of 148 enriched gene ontology categories found in postpriming conditions. GO enrichment analyses were done using a rank-based statistical test on log_2_ fold change of differentially expressed genes at each time compared to the control. A category was considered enriched under an FDR of <0.01. The intensity of the enrichment is expressed as the ratio between the number of genes that were significantly up (yellow) or down (blue) regulated in the category/total number of genes in the category. GO categories were clustered (1 to 6) according to the Pearson uncentered correlation (MeV_4_9_0 software). (D) Heat map focusing on the two clusters containing 27 immune-related GO categories (underlined in yellow) that were significantly enriched at 0.5 DPP, 1 DPP, or 1 DPC (FDR > 0.01, biological processes).

In order to identify the molecular basis underlying the resistance phenotype observed (i.e., acquired resistance to viral infection), we compared the transcriptomic responses of oysters after poly(I·C) priming or FSW injection at 0.5, 1, and 10 DPP. Illumina-sequenced reads were aligned to the *C. gigas* V9 reference genome (20); 74% of reads (on average) mapped on this genome (Table S2). We performed a technical validation of the RNA-seq approach by reverse transcription (RT)-qPCR on 20 genes at all sampling times (*r*^2^ = 0.7) (Fig. S1; Table S2). Differential expression of genes after poly(I·C) exposure was analyzed using the DEseq2 package through the comparison between poly(I·C) priming and FSW injection at each time point (0.5, 1, and 10 DPP). A total of 4,577 genes were significantly differentially expressed in at least one comparison (Fig. 4A; Table S3A). The number of differentially expressed genes (DEG) increased over time, ranging from 18% DEG early after priming (0.5 DPP) to 78% DEG at 10 DPP (Fig. 4B). Interestingly, a majority of these DEG were upregulated postpriming (from 83% at 1 and 0.5 DPP to 55% at 10 DPP [Fig. S2]).

10.1128/mBio.02777-19.1 FIG S1 Technical validation of the RNA-seq data by RT-qPCR. Twenty genes with contrasting expression levels were selected (the list of primers and PCR efficiencies are shown in Table S2), and their relative expression levels were quantified by RT-qPCR using the same RNA as used for the RNA-seq approach. These results, expressed as −ΔCq, were plotted against the log_2_ reads per kilobase per million (RPKM) obtained by the RNA-seq approach. The regression line (*y* = 1,0054*x* + 13,412) displaying *r*^2^ of 0.6843 indicates a good level of correlation between the two methods. Download FIG S1, TIF file, 1.7 MB. Copyright © 2020 Lafont et al. 2020 Lafont et al. This content is distributed under the terms of the Creative Commons Attribution 4.0 International license.

10.1128/mBio.02777-19.2 FIG S2 Distribution of differentially expressed genes postpriming. Numbers of DEG following poly(I·C) injection upregulated (red) or downregulated (green) are plotted following their fold change level. Download FIG S2, TIF file, 1.9 MB. Copyright © 2020 Lafont et al. 2020 Lafont et al. This content is distributed under the terms of the Creative Commons Attribution 4.0 International license.

10.1128/mBio.02777-19.7 TABLE S2 Primers used for RNA-seq validation and RT-qPCR analyses. Download Table S2, XLSX file, 0.02 MB. Copyright © 2020 Lafont et al. 2020 Lafont et al. This content is distributed under the terms of the Creative Commons Attribution 4.0 International license.

10.1128/mBio.02777-19.8 TABLE S3 (A) List of differentially expressed genes postpriming with poly(I·C); (B) DEG identification in primed oysters postchallenge. Download Table S3, XLSX file, 2.7 MB. Copyright © 2020 Lafont et al. 2020 Lafont et al. This content is distributed under the terms of the Creative Commons Attribution 4.0 International license.

These DEG were then submitted to a rank-based gene ontology analysis (RBGOA; false-discovery rate [FDR] < 0.01) to access the biological processes modulated following poly(I·C) priming. A total of 148 GO categories were significantly regulated in response to poly(I·C) at least one time (Fig. S3; Table S4). Intensity of enrichment of the 148 categories was represented in a heat map and hierarchically clustered (Fig. 4C). Six clusters were identified along the kinetic. We observed an increasing number of enriched functions from 0.5 DPP to 10 DPP. Numerous metabolic and cellular processes-related GO categories (regulation of RNA-DNA metabolic processes and modifications, carbohydrate metabolic processes, cell cycle regulation, organelle organization, protein modification, etc.) were found increasingly enriched (for both over- and underrepresented functions) along the priming kinetic (falling into clusters 3 to 6). The number of these categories related to metabolic and cellular processes represented 81% of all the enriched categories at 10 DPP, revealing a deep impact of the treatment on the long-term homeostasis and metabolism of the oyster (Fig. S3). Most interestingly, across all time points, we could find 31 enriched categories related to immune functions. Clusters 1 and 2 were highly enriched in these immune functions (27 immune categories, 68% of the 37 enriched categories found in clusters 1 and 2) revealing an immune response following poly(I·C) injection initiated from 0.5 DPP (where 78% of enriched categories were related to immune functions at that time point) and maintained through priming (74% of all of the immune function-related categories are still enriched at 10 DPP) (Fig. 4D). A majority of these categories were directly related to innate immunity and/or antiviral immunity, such as “innate immune response,” “regulation of cell death,” “Toll signaling pathways,” “response to virus,” and “regulation of type I interferon production,” likely to confer protection against subsequent viral infection. The genes behind these immune categories represent 54% of all the genes belonging to all the differentially enriched categories. This enrichment in immune categories strongly suggests that a systemic upregulation of many genes traditionally implicated in the response to viral infection occurred following poly(I·C) exposure and in the absence of virus.

10.1128/mBio.02777-19.3 FIG S3 Hierarchical clustering trees of Gene ontology categories affected by poly(I·C) priming. Hierarchical clustering trees of significant GO categories belonging to biological processes are shown. Font colors indicate enrichment of GO categories with either upregulated (red) or downregulated (blue) genes in oysters primed with poly(I·C) were compared to FSW at 0.5 days postpriming (A), 1 day postpriming (B), or 10 days postpriming (C). Font size indicates the level of statistical significance of the GO term calculated by the Mann-Whitney U test (*P* < 0.01, *P* < 0.001, or *P* < 0.0001). The key gives the correspondence of the fonts to significance thresholds. The fraction preceding the GO term indicates the number of genes significantly regulated in a category (numerator) and the total number of genes in this category (denominator). Branching indicates shared genes among GO categories. Download FIG S3, EPS file, 0.8 MB. Copyright © 2020 Lafont et al. 2020 Lafont et al. This content is distributed under the terms of the Creative Commons Attribution 4.0 International license.

10.1128/mBio.02777-19.9 TABLE S4 List of enriched categories identified by RBGOA. Download Table S4, XLSX file, 0.03 MB. Copyright © 2020 Lafont et al. 2020 Lafont et al. This content is distributed under the terms of the Creative Commons Attribution 4.0 International license.

### Poly(I·C) protects against OsHV-1 through a sustained immune response.

To understand the impact of poly(I·C) priming on antiviral protection, we analyzed the expression pattern of the genes associated with the enriched GO categories previously identified. We focused on the genes differentially regulated postpriming, since only 7 genes could be found to be significantly differentially regulated postchallenge when comparing (i) poly(I·C) primed oyster injected with the viral inoculum and (ii) primed oysters injected with a noninfectious inoculum (Table S3B). This result shows that only minor transcriptional changes occurred in primed oysters after challenge. They suggest that the acquired resistance phenotype does not need additional modifications after priming to maintain an antiviral barrier.

**FIG 5:**
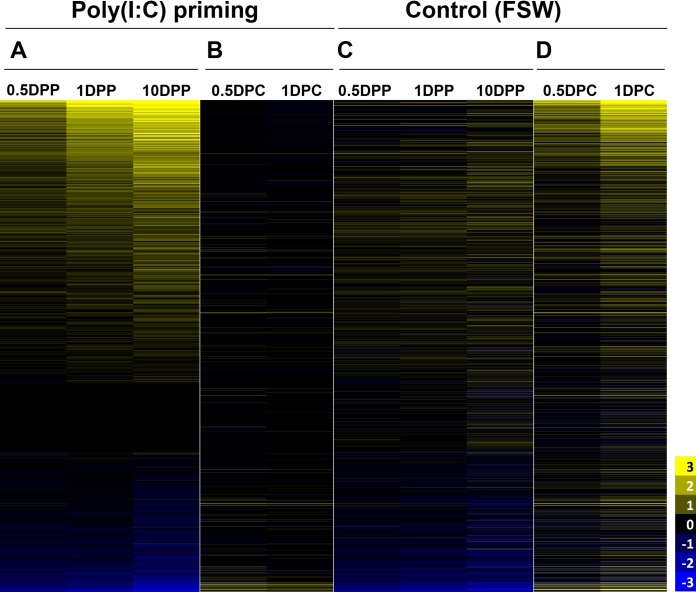
Poly(I·C) induces a massive upregulation of genes. The heat map shows expression kinetics of the 1,587 transcripts of oyster primed with poly(I·C) or injected with FSW as a control and challenged after 10 days with OsHV-1. The color scale indicates the log_2_ fold change calculated by DEseq2, of the 1,587 transcripts identified by RBGOA in comparing [poly(I·C) or FSW] 0.5, 1, and 10 DPP to T0 (FDR < 0.05) and 0.5 and 1 DPC to 10 DPP [poly(I·C) or FSW] (FDR < 0.05). The same transcripts are indicated in the same position for each condition. Heat maps for the poly(I·C) condition are shown in panels A and B which correspond to the postpriming and postchallenge time points, respectively. Heat maps for the control (FSW) condition are shown in panels C and D which correspond to the postpriming and postchallenge time points, respectively.

Excluding the “unknown” category, the remaining 147 enriched categories referred to 1,587 genes. To understand the molecular determinants behind priming, we first evaluated the expression rates of this set of genes by comparing the expression profiles of poly(I·C)-primed or FSW-injected oysters to unchallenged oysters (at the starting time of the experiment [T0]), along the postpriming kinetic (i.e., 0.5, 1, and 10 DPP). To understand the molecular determinants behind antiviral protection, we then compared the expression profiles at each time postchallenge (i.e., 0.5 and 1 DPC) to the 10-day postpriming condition (time zero of the challenge experiment) (Table S5A). For each comparison, the log_2_ fold change of significantly differentially expressed genes (FDR < 0.05) was plotted on a heat map. The results show that poly(I·C) priming induced a strong upregulation of gene expression (82% of the DEG) (Fig. 5A) as opposed to FSW-injected oysters (Fig. 5C; Table S5B). Moreover, poly(I·C)-primed oysters displayed an increasing number of regulated genes from 0.5 DPP to 10 DPP where 71% of the 1,587 genes were differentially expressed. Remarkably, following virus challenge, only a weak *de novo* regulation of oyster genes was observed in poly(I·C)-primed oysters (Fig. 5B) (only 13% of the 1,587 genes were differentially regulated) compared to the last priming time point (10 DPP). This is in contrast with FSW-injected controls, which showed a strong transcript upregulation (Fig. 5D) when facing the virus (62% at 1 DPC). This result shows that poly(I·C) treatment has major transcriptomic consequences: it induces a sustained response postpriming that is maintained postchallenge that can only be observed in animals protected against viral infection.

10.1128/mBio.02777-19.10 TABLE S5 (A) List of the 1,587 DEG regulated postpriming and their respective genome identifier, annotation, GO correspondence and FC, and RPKM in postpriming and postchallenge conditions; (B) number and percentage of regulated genes PP and PC among the 1,587 genes from enriched categories; (C) DEG expression pattern postchallenge; (D) comparison of DEG patterns between conditions; (E) identification of immune pathways within the DEG profiles. Download Table S5, XLSX file, 0.6 MB. Copyright © 2020 Lafont et al. 2020 Lafont et al. This content is distributed under the terms of the Creative Commons Attribution 4.0 International license.

**FIG 6:**
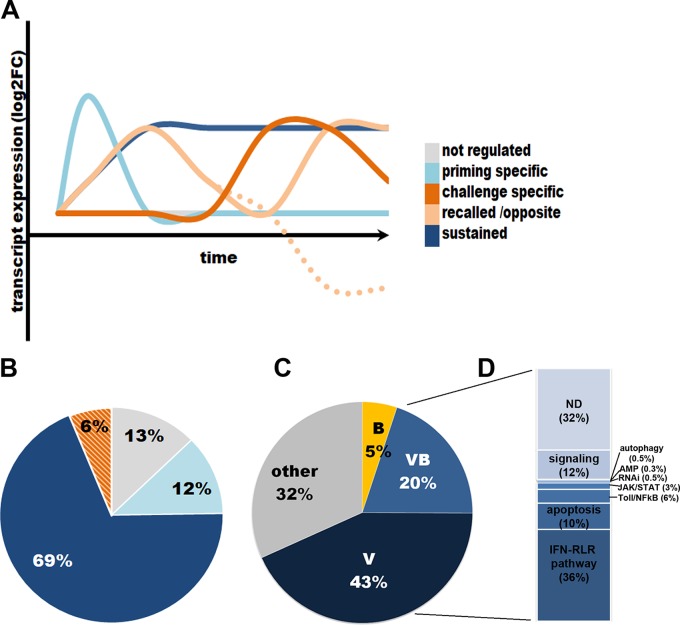
Regulation profile of transcripts from primed oysters after OsHV-1 challenge. (A) The expression profile followed by the 1,587 DEG could be categorized into 5 expression patterns: (i) “not regulated,” when genes are not regulated either postpriming injection or postchallenge compared to the reference times; (ii) “priming specific,” when genes are regulated after priming but not after challenge; (iii) “challenge specific,” when genes are regulated only after challenge; “recalled” (iv) and “recalled/opposite” (v), when genes are regulated in early times postpriming followed by a return to a basal state and regulated postchallenge, in the same or opposite way as postpriming; and (vi) “sustained,” when gene expression is maintained through priming and challenge (no return to a basal state). (B) Pie chart of the percentage of genes following the 5 different expression profiles regrouped into 4 (not regulated, regulated only after priming, regulated after challenge regrouping challenge specific and recalled/opposite patterns, and sustained) in poly(I·C)-primed oysters after OsHV-1 challenge. (C) Pie chart of the percentage of genes from the poly(I·C)-primed condition with a sustained expression profile (without genes also sustained in nonprimed oysters) that have been categorized as associated with an antibacterial (B), antiviral (V), or antimicrobial (VB) or not associated with any treatment (other). (D) Chart of the percentage of genes from the V and VB categories categorized associated with immune pathways. ND, not determined (genes could not be associated with a known immune pathway); AMP, antimicrobial peptide.

The analysis of the regulation profiles following priming and virus challenge allowed us to identify 6 different gene expression patterns (Table S5C): “not regulated”; genes regulated only after priming (“priming specific”) or after challenge (“challenge specific”); genes regulated after priming, followed by a return to a basal state (extinction of the signal) and a regulation after challenge (“recall” and “recall/opposite”); genes regulated postpriming whose expression is maintained throughout priming and challenge with no extinction of the signal (“sustained”). On one hand, analyses of the number of genes falling into each pattern confirmed that the main expression profile in poly(I·C)-primed oysters was a sustained response (69%) (Fig. 6B). On the other hand, controls presented a large set of genes upregulated after challenge (challenge specific and recall/opposite; 43%) besides a sustained pattern (40%). The overall expression pattern in poly(I·C)-primed oysters is then different from that of the control, with a main sustained response for the primed oysters and a main regulation after virus challenge for the controls.

### Antiviral pathways support protection against OsHV-1.

To get a deeper insight into the immune determinants involved in the antiviral protection observed, we then analyzed the putative functions of the genes falling into the different expression profile. We assigned a function to each gene following a BLAST (Basic Local Alignment Search Tool) analysis using known databases and comparison with the literature. We focused on the genes that followed the main expression pattern in response to poly(I·C), i.e., a sustained expression profile. We restricted this analysis to genes specific to poly(I·C) response in excluding genes whose expression was also found sustained under the control condition [Table S5D]). We found that 43% of those genes could be assigned to an antiviral response (“V”), 5% to an antibacterial response (“B”), and 20% potentially to both (“V/B”), while 32% could not be directly associated with known immune pathways (“other”) (Fig. 6C).

In the sustained expression profile, we then assigned the genes from the “V” and “V/B” categories to known antiviral pathways (Fig. 6D; Table S5E). This analysis revealed important contributions of immune-related multigene families and of the main recognized antiviral pathways. The multigene families containing 5 to 51 different genes each included genes belonging to known antiviral pathways and apoptosis (RNA helicase, SAMHD-1, E3 ubiquitin ligase, IFI44, ADAR, Toll-like receptor [TLR], sacsin, STING, IRF, interferon [IFN] alpha-inducible proteins, tripartite motif protein [TRIM], cGAS, IAP, tumor necrosis factor receptor [TNF-R], caspases, and Fas ligand) or more pleiotropic immune pathways (C1q, peroxidase, macrophage mannose receptors and lectins, and NFX1). Hence, a number of genes could be associated with the Toll/NF-κB and the JAK/STAT pathways that are tightly linked to the IFN pathway. The IFN pathways (36% of the genes) include RLR and STING-dependent pathways from receptors (RLR, TLR, and cGAS) to interferon-stimulated genes and antiviral effectors (e.g., ADAR, IFI44, viperin, and sacsin). The second most represented group of genes belongs to the apoptosis pathway (TNF, Fas, caspases, and IAP). Interestingly, a majority (79%) of postchallenge regulated genes in control oysters were also found in the sustained profile from primed oysters (Table S5D; Fig. S4A). A close analysis of the common DEG in the poly(I·C) sustained profile and the control challenge-specific profile confirmed that they followed the same expression pattern, i.e., that they were mainly upregulated, with similar levels of fold change (Fig. S4B). Moreover, comparative analysis of the putative functions of these genes also showed that genes involved in the challenge response in the FSW controls could be assigned in the same proportions as poly(I·C) sustained DEG to antimicrobial functions (mainly antiviral) and antiviral pathways (mainly IFN-like pathways) (Fig. S4C and D). Together, these results illustrate that similar sets of genes seem to be involved (i) in protecting primed oysters from the virus and (ii) the inefficient antiviral response of nonprimed oysters.

10.1128/mBio.02777-19.4 FIG S4 Comparative analysis of the poly(I·C) sustained expression pattern and the FSW-specific challenge. (A) Venn diagram representing the genes common to the two patterns. (B) Comparison of the percentage of DEG within each pattern. Different shades of red represent fold change categories of upregulated genes (>0, >2, and >5). Different shades of green represent fold change categories of downregulated genes (>0, >−1, and >−2). (C) Comparison of the number of genes falling into the virus (V), virus/bacteria (V/B), bacteria (B), and other categories. (D) Comparison of the numbers of genes falling into different immune-related gene categories. Download FIG S4, TIF file, 1.0 MB. Copyright © 2020 Lafont et al. 2020 Lafont et al. This content is distributed under the terms of the Creative Commons Attribution 4.0 International license.

### The poly(I·C)-induced antiviral response is maintained over 4 months.

**FIG 7:**
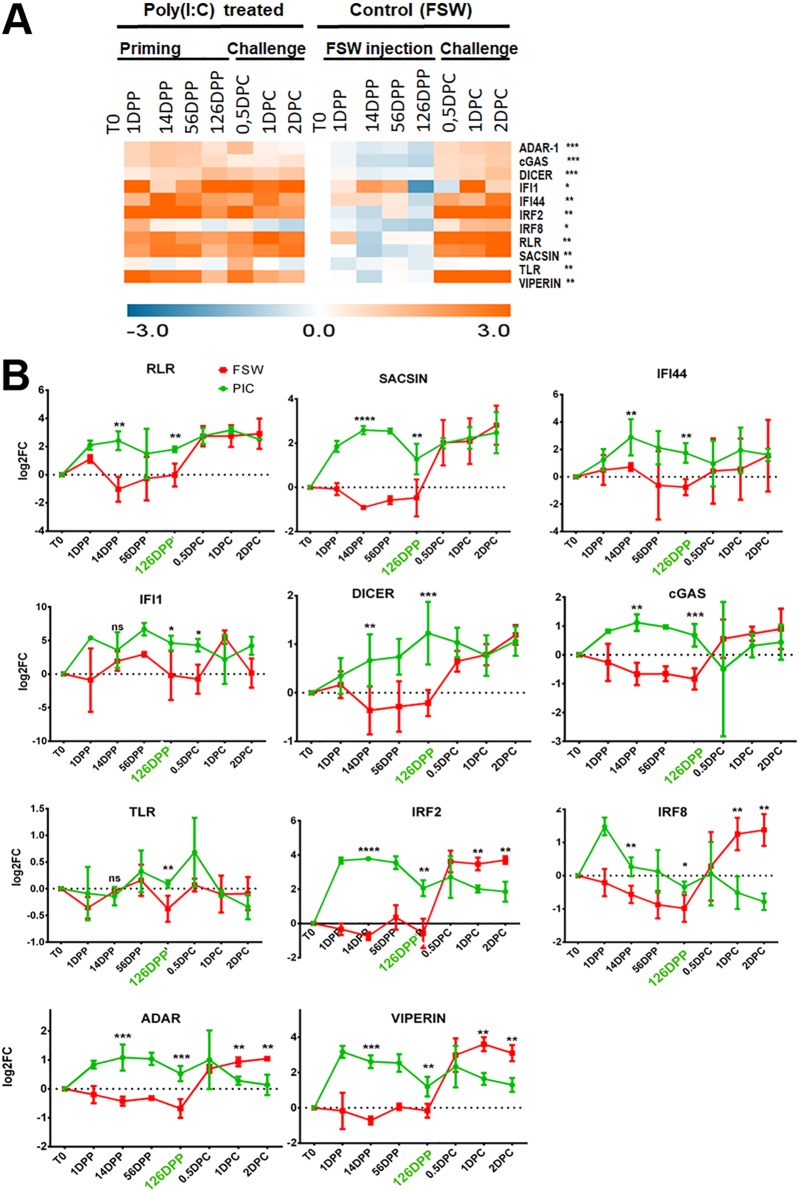
Gene expression pattern of a long-term priming experiment. The expression pattern of 11 candidate genes was followed in oysters primed with poly(I·C) or injected with FSW as a control and challenged after 126 days with OsHV-1. (A) Heat map generated from the log_2_ fold change (FC) of the 11 genes at 1, 14, 56, and 126 days postpriming and 0.5, 1, and 2 days postchallenge. Log_2_ FC was calculated by the 2^−ΔΔCq^ method in comparing each time postpriming and postchallenge to T0. Genes from primed oysters with FC significatively different from the control (FSW) at 126 DPP are indicated by asterisks (multiple *t* test; *, *P* < 0.1; **, *P* < 0.5; ***, *P* < 0.001). (B) Graph plotting the log_2_ FC of the 11 genes over the kinetic of priming and challenge. Asterisks indicate significant differences between transcripts’ expression at 14 or 126 days postpriming and time zero (multiple *t* test; *, *P* < 0.1; **, *P* < 0.5; ***, *P* < 0.001; ****, *P* < 0.0001).

To determine whether the protective antiviral response could be responsible for long-term protection, we next monitored the expression of 11 candidate genes from antiviral pathways whose expression was found sustained on a 10-day priming kinetic (Fig. S5; Table S2). For that, we used a former sampling in which poly(I·C) was shown to confer antiviral protection from 24 h to 4 months (126 days) after priming (16). Analyses performed over 126 days revealed that all genes followed the same expression pattern as on a 10-day priming kinetic, with a significant upregulation from 1 day to 126 days postpriming, as opposed to nonprimed conditions, under which genes were strongly upregulated only after challenge (Fig. 7A). All genes were still significantly upregulated at 126 days postpriming (Fig. 7A). Although upregulation tended to decline throughout the kinetic, significant differential expression was still observed compared to that with the controls (Fig. 7B). Those genes do not seem to be further regulated postchallenge. However, they follow two different patterns. A first set of genes shows a sustained upregulation that will be maintained at the level of nonprimed oysters’ antiviral response (RLR, sacsin, IFI44, IFI1, DICER, cGAS, and TLR). A second set of genes shows a sustained response that will be significantly lower than in the control where the response to the virus induces a stronger upregulation (IRF2, IRF8, ADAR, and viperin). Together, these results suggest that a long-term sustained response relies on an increased level of immune activation.

10.1128/mBio.02777-19.5 FIG S5 Experimental design used to identify the long-term molecular basis underlying poly(I·C) priming. (A) Specific-pathogen-free (SPF) oysters, highly susceptible to juvenile oyster syndrome, were anesthetized before being primed with poly(I·C) or filtered seawater (FSW) as a control. Oysters were sampled for RNA extraction and gene expression analysis at 1, 14, 56, and 126 days after priming. At 126 days postpriming (DPP), oysters from each condition were challenged with OsHV-1 inoculum (1.22 × 10^8^ copies of DP gene μl^−1^) or OsHV-1-free inoculum (control). Survival of oysters was monitored for 10 days postchallenge (DPC). Three pools of 3 oysters for each condition were sampled postpriming and postchallenge (0.5 day and 1 day) for viral load analyses or RNA sequencing. (B) Kaplan-Meier survival curve of the experiment. Mortalities in each group of 45 oysters (15 per tank) were monitored for 10 days after infection. a and b, *P* value < 0.0001 (log rank test; *n* = 45). Conditions demonstrating 100% survival rates can appear hidden and merged with other ones reaching the same survival rate. One control condition without injection is represented as nontreated (black line). (C) OsHV-1 DNA detection by quantitative PCR. Results are expressed as the mean number of DP copies detected per nanogram of genomic DNA extracted from whole spat pools at the different times postchallenge. Statistically significant differences were found using multiple *t* test for the FSW plus OsHV-1 condition at 6 DPC (*P* value < 0.0001) compared to T0 of the challenge; *n* = 3 pools of 3 oysters. Download FIG S5, TIF file, 0.1 MB. Copyright © 2020 Lafont et al. 2020 Lafont et al. This content is distributed under the terms of the Creative Commons Attribution 4.0 International license.

## DISCUSSION

We characterized the molecular basis of antiviral immune priming conferring long-term resistance to Pacific oyster mortality syndrome in *Crassostrea gigas*. Immune priming was induced by exposure to poly(I·C), which was earlier characterized for its major protective effect against OsHV-1 infection, both under controlled experimental conditions and in field experiments (15, 16).

As in earlier experiments, poly(I·C) injection was very efficient in increasing to 100% the survival of the oyster and reverting to resistant the phenotype of an oyster family genetically susceptible to POMS (14). In this study, we could access the molecular basis underlying this drastic phenotypic change through a global transcriptomic analysis of the oyster response to poly(I·C) and subsequent viral infection. The main response observed in our experiment was a sustained upregulation of gene expression. Remarkably, only minor transcriptional changes occurred in primed oysters after viral infection. This response was persistent over time and was still observed 4 months after priming, although it remains unclear whether such a primed defense response may last longer, which should be investigated in the future. Interestingly, we previously showed that poly(I·C) was short-lived in the oyster hemolymph, which strongly suggests that the stimulus is long gone and has ceased a direct effect and that the priming information has been stored (16). This long-term active state of immune genes fits with a sustained immune regulation scenario, which has been proposed as the main immune priming response in many invertebrate species, based solely on the observation of long-lasting antibacterial activities (antimicrobial peptide activities, phenoloxidase, and phagocytic activities) (21). At the transcriptomic level, this immune alert state has been described only in a priming context for an insect, after a 7-day priming period (22). But it differs from previous transcriptomic analyses in invertebrates, in which the main priming response observed was an immune shift (17, 19). In addition, this transcriptomic modification of the gene basal state has been reported in other defense contexts, where it was shown to increase immune competence and resistance status in plants, insects, and fish (23–27). The observation of an elevated basal level state of immune genes could also be interpreted as a decrease in transcriptomic plasticity, a mechanism known as gene expression frontloading and described as an adaptive mechanism (28, 29).

From our study, this preinduction of immune genes appears to be an efficient mechanism to provide rapid protection against viral replication. Interestingly, the immune repertoire activated upon priming did not seem to qualitatively differ from the one mounted upon infection in nonprimed oysters (79% in common). These results illustrate that a similar set of genes might be involved in response to poly(I·C) in primed oysters and to the virus in the controls but was inefficient in protecting the oyster in the latter case. This suggests that the implementation of an efficient antiviral response in the oyster is highly dependent on the timely activation of antiviral mechanisms. This is highly consistent with our earlier observations comparing oysters susceptible or resistant to POMS. Indeed, we showed that resistant oysters could mount an early antiviral response sufficient to control viral replication, while the late immune response of susceptible animals led to a fatal disease outcome (14). Together, these two recent studies converge in illustrating Graham and Tate’s conclusion that the sooner the immune system launches, the greater the chances the host has of survival (30). It is also consistent with recent systems biology approach suggesting that early pathogen/host encounter could influence the final outcome of many infections (31). We found that by reducing time response to infection to zero, immune priming prepares the host to face the infection, and the virus does not replicate anymore in the host. That elevated basal state could prophylactically confer a survival benefit with larger amounts of immune effectors that would be “ready-to-use” upon subsequent challenge that could be a form of immune conditioning. This strongly suggests, as exemplified for insects (32), that the probability of survival of oysters to POMS could be predicted on the basis of its immune state prior to infection.

In this study, this acquired antiviral alert state has been associated with conserved key antiviral pathways contributing to a rapid defense response. An IFN-like pathway (from receptors to interferon-stimulated genes and antiviral effectors) was activated in response to the priming. Consistently, numerous regulated genes could also be assigned to genes from the Toll/NF-κB pathway, well known for its involvement in antiviral response and cross talk with the IFN pathway in mammals. Apoptosis appeared as the second most represented category among differentially regulated genes. Apoptosis mechanisms have been well documented in many models, as in oysters, to be a key antiviral response associated with enhanced viral protection as well as a target of virus to counteract antiviral defenses (14, 24, 33, 34). This observation suggests that apoptosis might be associated with enhanced viral protection, possibly by eliminating virus-infected cells at an early stage of infection as a rapid mechanism of defense against the virus. JAK-STAT and RNA interference (RNAi) pathways, hallmarks of the antiviral response in insects, were also identified (35). Altogether, the set of genes found to be activated upon priming or challenge is very consistent with previous studies regarding immune gene regulation following poly(I·C) in mammals and arthropods or OsHV-1 infection in oyster (14, 15, 36–44). Previous studies reported the massive expansion of some immune-related gene families in oysters (36, 45) and in antiviral response (46, 47). This study also highlighted the implication of numerous previously described immune multigene families, which is especially true for recognition receptors (11, 36, 45, 47–49). This abundant gene polymorphism, allowing higher variability, could play a role in mediating this priming response.

How permanent changes in host immune gene expression are achieved is still an open question. Little is known for the oyster and more generally for invertebrates, which lack the classical adaptive immune system (lymphocyte and immunoglobulin-related), regarding the cellular and molecular carriers of immune priming. Epigenetic changes seem to be a prerequisite for trained innate immunity in mammals and a convergent feature of innate immune memory (50, 51). Epigenetic changes in the vicinity of the promoters of candidate immune genes could explain, at least partially, how the baseline transcript expression is lastingly changed in response to priming. Interestingly, among the 1,587 DEG identified, we could find 67 genes which GO terms or sequence homologies that could be related to the epigenetic machinery (GO terms responding to keywords as epigenetic, methylation, methyltransferase, histone methylation-acetylation-modification, chromatin modification-remodeling) (52–55) (Table S5A). In addition, there is a growing body of literature showing new results about the intricate interplay between epigenetic regulation and immunity. For example, one study (56) recently showed that STATs had critical roles early in infection and promoted early epigenetic and transcriptional changes; they may have diverse stage-specific roles that cumulatively optimize proper memory formation and maintenance. The rapidly growing field of epigenetic studies should allow us to further investigate in the near future if epigenetic mechanisms support oyster immune priming and whether it can target specific immune pathways.

Finally, if antiviral immune priming appears as an attractive option to protect oysters from POMS, it is important to investigate the physiological cost of investment in immune priming. Persistent immune protection and an overall increased capacity of host defense should be beneficial in long-lived organisms, likely to face repeated exposures to same or similar pathogens. But this investment may have damaging effects if activated at the wrong time and in an inflated fashion. In our study, the identification of enriched categories (positively or negatively enriched) associated with metabolic and cellular processes could also be indicative of a detrimental impact of priming on the oyster fitness and/or involve more than strictly immune function to achieve protection. Interestingly, interplays between metabolic reprogramming and immunity have been evidenced as an ancestral principle that could support innate immune priming in very distant organisms such as C. elegans and mammals (51, 57). Previous studies have also already emphasized the potential trade-offs linked to innate immune priming notably on reproduction potential (58–60) but also between immunity and physiological status in the oyster (61). Interestingly, we observed that the elevated immune baseline in long-term priming did not systematically result in a strong secondary response when facing viral infection. This could be suggestive of a modified inflammatory state that would limit the detrimental effects of a persistent immune response. In addition, no impact on oyster survival could be identified over a 4-month priming period in our setting. This trade-off issue should, however, be considered in future studies to address the cost of maintaining a primed immune response and the beneficial versus potential detrimental effects of the protection. In this regard, our study could help identify priming markers that could be used to follow the immune state of oysters in the wild. Evaluating their “resistance/primed” status due to a previous encounter with OsHV-1 could help evaluate the impact of priming on oyster fitness and adaptability *in natura*. Such insights on the impact of long-term immune priming on oyster physiology and host-pathogen dynamics are essential to develop strategies to mitigate impacts of recurrent pathogen exposure (60).

In conclusion, we reveal here a novel aspect of immune priming through a modified constitutive level of defense gene expression allowing effective adaptation to reinfection. This persistent antiviral state was notably supported by the upregulation of a large array of antiviral pathways conserved from vertebrates, insects to the oyster (IFN, TLR/NF-κB, JAK/STAT, and apoptosis), and multigene families. This response differs from previous observations from invertebrates in that it is very long-lasting and relies mainly on a sustained response. These discrepancies in molecular underpinnings of invertebrate’s immune priming further suggest that, as previous authors stated (62), innate immune memory can be “multiply realized” in different animal systems and should not be restricted to one mechanistic model. The present study further warrants the need for in-depth analysis of molecular basis of immune priming to compare mechanisms supporting these convergent phenomena throughout animal phyla. Further integrated (transcriptomic, metabolic, cellular, and epigenetic) analyses could help understand whether protection is dependent on coordinated metabolic and transcriptional control as well as the cellular support of this memory. This antiviral immune priming of oysters represents an alternative strategy that holds promises to increase the capacity of oysters to cope with current biotic stresses and to prevent, at long last, Pacific oyster mortality syndrome, one of the most devastating diseases affecting shellfish.

## MATERIALS AND METHODS

### Preparation of viral and control inocula.

OsHV-1 inocula were prepared according to reference 63 from moribund oysters experimentally infected with OsHV-1 in previous trials and frozen at –80°C. Viral DNA loads (OsHV-1 genome copies per microliter) in inocula were estimated by quantitative PCR (qPCR) (see “Viral detection and quantification” below). Control inocula were prepared following the same protocol from healthy oysters showing no detectable amount of viral DNA. Inocula were confirmed to be free of cultivable bacteria by plating 40 μl on LB-NaCl agar plates.

### Priming experiments.

**(i) Experiment for RNA-seq analysis.** The experiment for RNA-seq analysis was performed with specific-pathogen-free juveniles (SPF; i.e., free of mortality, negative for OsHV-1 detection, and very low concentrations of *Vibrio* bacteria [64]) (3.5 months; shell length, 20.6 ± 0.1 mm; total weight, 1.62 ± 0.1 g; means ± standard errors [SE]) of the Pacific oyster *Crassostrea gigas* (or Magallana gigas [65], Thunberg 1793). Oysters were offspring of the F_11_ family of the DECIPHER program (ANR-14-CE19-0023) produced in July 2016. These oysters were chosen for their high susceptibility to juvenile oyster syndrome (14).

As priming solution, poly(I·C) high molecular weight (HMW) (InVivogen; catalog code tlrl-pic) was prepared following the manufacturer’s instructions and diluted in filtered seawater (FSW) to a concentration of 475 ng μl^−1^. As a control, a solution of autoclaved and filtered seawater at 0.02 μm was used. For the priming experiment, oysters were anesthetized in hexahydrate MgCl_2_ (ACROS; catalog number 197530250, 50 g liter^−1^, 100 oysters liter^−1^) according to the method of Suquet et al. (66) for 8 h. Animals were then primed by injection of 40 μl of priming solutions in the adductor muscle, using a 26-gauge needle attached to a multidispensing hand pipette. A group of oysters was kept untreated during all the experimentation (i.e., not primed or challenged). Oysters were reared in seawater at 15 ± 1°C for 10 days. For challenge, oysters were reanesthetized and injected with 20 μl of OsHV-1 or control inoculum (same injection method). Each challenge was performed in 4 replicates in 5-liter seawater plastic tanks regulated at 20 ± 1°C. In each tank, oysters were separated in two groups: one for survival monitoring (15 oysters) and one for sampling (75 oysters). Survival was followed daily and dead oysters were removed from tanks. Survival rates were analyzed for statistical differences between treatments by log rank test on Kaplan-Meier survival curves using the computer software package GraphPad Prism v. 6.0. Sampling of pools of 15 animals was performed at time zero (T0), at 12 h (0.5 days), 1 day, and 10 days postpriming (DPP), and at 0.5, 1, 2, and 7 days postchallenge (DPC). Samples from T0, 0.5, 1, and 10 DPP, 0.5 and 1 DPC were used for DNA and RNA analyses; other samples were used for DNA analyses only (Fig. 1).

**(ii) Long-term poly(I·C) protection.** The experiment was performed as described previously (16). For each priming condition (Fig. S5), 1,200 spats from the 01–2016 FINA cohort (Ifremer production) were injected after anesthesia with poly(I·C) HMW (19 μg · g^−1^ of oyster) and 1,200 spats were injected with filtered seawater as a control on the same day. At 126 days after priming, a group of oysters (120 spats) was infected with OsHV-1 (1.22 × 10^8^ copies of DP gene μl^−1^) or control inocula (120 spats) to test if the poly(I·C) treatment was still protecting the oysters from OsHV-1 infection. Sampling of 3 pools of 3 oysters was performed at each time point postpriming (0.5, 1, and 10 DPP) and postchallenge (0.5 and 1 DPC) for each condition.

### Sample treatment.

For all experiments, sampling consisted of individually removing shells of each animal with a sterile scalpel blade and snap-freezing the whole oyster tissue in liquid nitrogen. Samples were stored at –80°C before grinding and homogenization by bead beating (Retsch; Mixer Mill MM400) with a 20-mm stainless steel ball in 50-ml stainless steel bowls prechilled in liquid nitrogen. Powders obtained were then stored at –80°C and used for DNA and/or RNA extractions.

### Extraction of nucleic acids.

Genomic DNA was purified from homogenized oyster powders using the Macherey-Nagel genomic DNA-from-tissue kit (catalog number 740952.250) following the manufacturer’s instructions, with an additional step of RNase A treatment (Macherey-Nagel; catalog number 740505). The quality and quantity of genomic DNA were estimated on a NanoDrop ND-1000 spectrophotometer (Thermo Scientific). Total DNA was resuspended to a final concentration of 20 ng μl^−1^ prior to qPCR. Total RNA was extracted from oyster pool powders (10 mg) homogenized in 500 μl of Tri-Reagent (Invitrogen) by vortexing for 2 h at 4°C. Prior to extraction, insoluble materials were removed by centrifugation at 12,000 × *g* for 10 min at 4°C, and supernatant was incubated with 100 μl of chloroform at room temperature for 3 min. After centrifugation at 12,000 × *g* for 15 min at 4°C, total RNA in the aqueous phase was extracted using the Direct-Zol RNA miniprep kit (Zymo Research; reference no. R2052) according to the manufacturer’s protocol. Quantity and purity of total RNAs were checked using the NanoDrop ND-1000 spectrophotometer (Thermo Scientific) and capillary electrophoresis (Agilent BioAnalyzer 2100).

### Viral detection and quantification.

Detection and quantification of OsHV-1 DNA were performed using quantitative PCR (qPCR). All amplification reactions were performed in duplicate using a Roche LightCycler 480 real-time thermocycler (qPHD-Montpellier GenomiX platform; Montpellier University). PCR volumes were 6 μl, containing LightCycler 480 SYBR green I master mix (Roche), 100 nM pathogen-specific primers, and 20 ng of DNA. PCRs were performed using an initial denaturation (95°C for 10 min) followed by 40 cycles of denaturation (95°C for 10 s), hybridization (60°C for 20 s), and elongation (72°C for 25 s). Virus-specific primers targeting a region of the OsHV-1 genome predicted to encode a DNA polymerase catalytic subunit (DP) were chosen as previously described (ORF100; nucleotides 147655 to 153291 of the OsHV-1 genome [GenBank accession number AY509253]: OsHVDPFor, ATTGATGATGTGGATAATCTGTG; OsHVDPRev, GGTAAATACCATTGGTCTTGTTCC) (67). For absolute quantification, DP amplification products were cloned into the pCR4-Topo vector and replicated in Escherichia coli DH5α (Invitrogen). Plasmids were extracted using the Wizard Plus SV miniprep DNA purification system (Promega) and standard curves of known concentration of plasmid generated according to the Applied Biosystems manual of absolute real-time RT-PCR quantification (77). Absolute quantification of OsHV-1 genome copies in oyster samples was then estimated by comparing the observed Cp values to known plasmid standards from 10^3^ (limit of detection) to 10^9^ copies of DP. Viral load data, expressed in copies of DP per nanogram of total gDNA, were transformed with the neperian logarithm function y=ln(x+1) and normality (D’Agostino and Pearson omnibus normality test, *P* value < 0.05) and variance homoscedasticity (Brown-Forsythe test, *P* value < 0.05) were tested. The effect of priming on OsHV-1 load was determined using one-way analysis of variance (ANOVA) using the computer software package GraphPad Prism version 6.0. Bonferroni’s correction for multiple-comparison tests was used to compare the viral load from each postchallenge sample to 10 DPP.

### RNA sequencing.

Quality check of total RNA was performed by capillary electrophoresis (Agilent BioAnalyzer 2100) and showed no degradation. mRNA purification and library construction were performed by MGX (Montpellier Genomix, Institut de Génomique fonctionnelle, Montpellier, France). Libraries were constructed using the Truseq stranded mRNA sample prep kit (Illumina; RS-122-2101) according to the manufacturer’s instructions. Briefly, poly(A) RNAs were purified using oligo(dT) magnetic beads. The poly(A)^+^ RNAs were fragmented and reverse transcribed using random hexamers, SuperScript II (Life Technologies; 18064-014) and actinomycin D. During the second-strand generation step, dUTP was replaced with dTTP. This prevents the second strand from serving as a template during the final PCR amplification. Double-stranded cDNAs were adenylated at their 3′ ends before ligation was performed using Illumina’s indexed adapters. Ligated cDNAs were amplified following 15 cycles of PCR, and PCR products were purified using AMPure XP beads (Beckman Coulter Genomics; A63881). Libraries were validated using a Fragment Analyzer (Agilent) and quantified using the KAPA library quantification kit (Roche; KK4824). Equimolar pools including 5 or 6 libraries were generated, and sequencing was performed on a HiSeq2500 using the single-read protocol (50 nucleotides [nt]) on a total of 9 lanes using v4 reagents.

### RNA-seq analysis.

All data treatments were carried out on a local Galaxy server (https://bioinfo.univ-perp.fr) (68). Read quality was checked by FastQC software (version 0.11.5), and the high quality observed (Phred score >32) allowed us to keep all sequenced reads for further analyses. The sequencing depth was between 19 and 40.3 million reads per sample (Table S1).

For OsHV-1 transcriptome analysis, reads were aligned to the genomes of OsHV-1 variant A (GenBank accession number KY242785) and variant B (GenBank accession number KY271630) (69) using Bowtie2 (Galaxy version 2.3.0.1) (70) (Table S1). The read count was performed using HTSeq-count (Galaxy version 0.6.1) (71). For each sample, each open reading frame (ORF) at each time, the read count number was normalized (NC) accordingly to the following formula:

$$\text{NC}=\left(\frac{C_{\text{ORF}}}{S_{\text{ORF}}}\right)/\left(\frac{N_{\text{reads}}}{N_{m}}\right)$$

where *C*_ORF_ is read count, *S*_ORF_ is size of the ORF in kilobase pairs, *N*_reads_ is the total number of reads sequenced for a sample, and *N_m_* is the mean of all reads sequenced for all samples (=37.40 million reads).

For each sample, all normalized read counts for all ORFs were added up. The effect of priming on replication during OsHV-1 challenge was determined using one-way ANOVA as described in the “Viral detection and quantification” section. Bonferroni’s correction for multiple-comparison test was used to compare the NC from each postchallenge sample to that at 10 DPP [poly(I·C) or FSW].

For oyster transcriptome analysis, mapping on the *C. gigas* reference genome (version V9 [20]; (GenBank accession number AFTI00000000) was performed using RNA STAR (Galaxy version 2.4.0d-2) under default parameters (72). The read count was performed using HTSeq-count (Galaxy version 0.6.1), in union mode, with a minimum alignment quality of 10 and feature type of mRNA (71). The gff file used as input was provided by Zhang et al. (20). DEseq2 (Galaxy version 2.11.39) was used to analyze the differential gene expression level between treatments (73). Fold changes between treatments were considered significant when the adjusted *P* value with the false-discovery rate (FDR) (Benjamini-Hochberg procedure [74]) was <0.05.

### Gene ontology analysis.

In order to identify biological functions affected by poly(I·C) priming, enrichment analyses were performed by a gene ontology (GO) analysis using a rank-based statistical test (Mann-Whitney U test) combined to an adaptive clustering (75). Analyses were done with an R and Perl script available at https://github.com/z0on/GO_MWU (76). The following parameters were used: largest = 0.1, smallest = 10, and clusterCutHeight = 0.25. To generate the table of continous measures, from the 28.027 genes of the oyster genome, the log_2_ fold change was assigned to genes significantly differently expressed (DEseq2; FDR < 0.05) and “0” to genes that were not significantly differentially expressed. A GO category was considered enriched for an FDR of <0.01. To synthetically represent the GO results, a ratio was calculated for upregulated and downregulated enriched categories with the following calculations: (i) for upregulated enriched categories, (number of genes significantly upregulated in the category/total number of genes in this category) or (ii) for downregulated enriched categories, −1 × (number of genes significantly downregulated in this category/total number of genes in this category). Results were represented on a heat map using a hierarchical clustering with uncentered Pearson correlation (MeV 4_9_0 software).

### Analyses of transcript differential expressions along the priming and challenge kinetics.

In order to characterize the effect of priming and challenge on the expression pattern of candidate genes through time, DEseq2 was used to calculate log_2_ fold change expression on the set of genes present in the enriched GO categories previously obtained. Regarding postpriming [poly(I·C) or FSW], each time point (i.e., 0.5, 1, and 10 days) was compared to T0. Regarding postchallenge with OsHV-1, each time point (i.e., 0.5 and 1 day) was compared to 10 DPP. To precisely determine the effect of OsHV-1, a third DESeq2 analysis was performed postchallenge by comparison between OsHV-1 and control inoculum treatments under poly(I·C)- and FSW-primed conditions. Results were represented on a heat map using Microsoft Excel 2010. To investigate the putative functions of the genes falling into the different expression patterns, we used the functional annotations of those genes. To work with current functional annotations of the *C. gigas* gene set, we performed a *de novo* functional annotation. BLASTX comparison against the NR database was performed for the 28,027 genes annotated in the genome, with a maximum number of target hits of 20 and a minimum E value of 0.001. The putative role of immune genes was assessed by investigating related literature.

### RT-qPCR analyses.

Total RNA (1 μg) extracted from ground oyster powder was reverse transcribed in 20 μl using the Moloney murine leukemia virus reverse transcriptase (MMLV-RT; Invitrogen) according to the manufacturer’s instructions using random primers (Invitrogen). Pipetting and amplification into 384-well plates were performed with a Labcyte acoustic automated liquid handling platform (ECHO) and a Roche LightCycler 480, respectively. The total RT-qPCR volume was 1.5 μl and consisted of 0.5 μl of cDNA (dilution, ¼) and 1 μl of LightCycler 480 SYBR green Master I mix (Roche) containing 0.5 μM PCR primers (Eurogentec SA; primer list in Table S2). The amplification efficiency of each pair of primers was validated by serial dilution of a pool of all the cDNAs. Relative expression quantification was calculated according to the quantification cycle (2^−ΔΔCq^) method with the mean of two reference genes (*Cg*-RPL40, GenBank accession number FP004478; *Cg*-RPS6, GenBank accession number HS119070).

### Statistics.

Statistical data analysis was carried out in GraphPad Prism (version 6.0) for Windows (GraphPad Software, La Jolla, CA). For group comparisons, multiple *t* tests were performed. Survival rates are represented as Kaplan-Meier curves.

### Data availability.

RNA sequence raw data are accessible on the Ifremer database with the following link: https://doi.org/10.12770/78b72405-f1ca-44a5-ae15-65916de3b2dd.
