# Supplementary figures and images for: A Sustained Immune Response Supports Long-Term Antiviral Immune Priming in the Pacific Oyster, Crassostrea gigas
Source: mBio. 2020 Mar 10;11(2):e02777-19. doi: 10.1128/mBio.02777-19 (PMC7064767; doi:10.1128/mBio.02777-19)

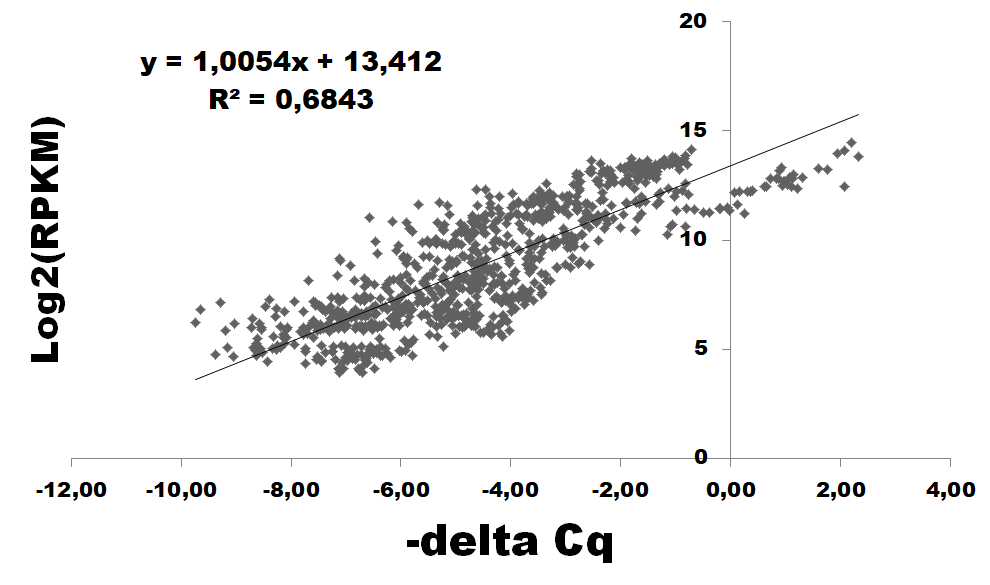

Supplement: FIG S1 [file mBio.02777-19-sf001.tif]

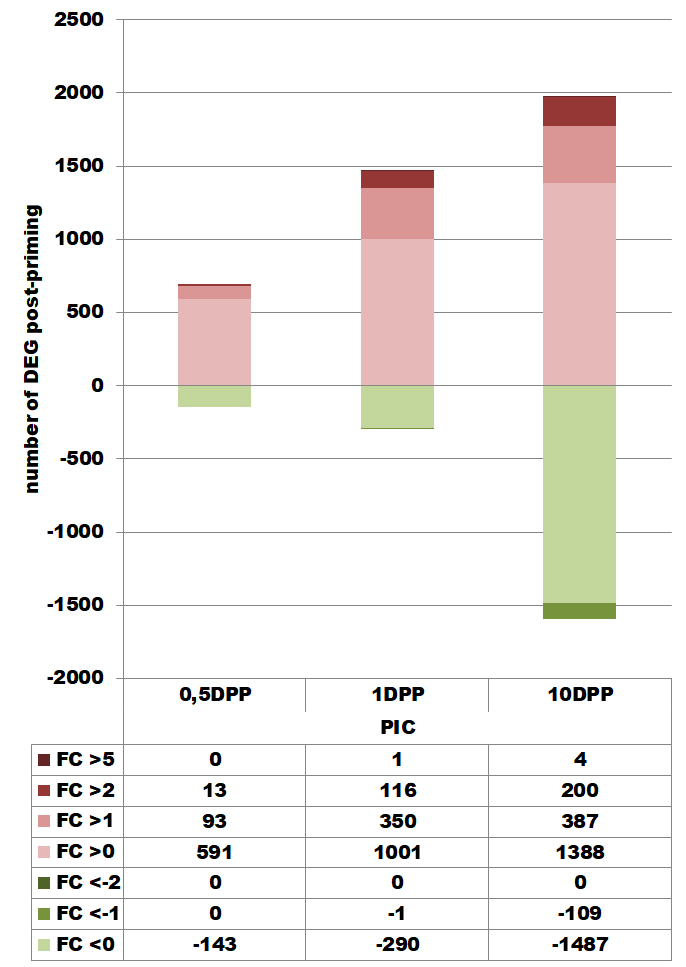

Supplement: FIG S2 [file mBio.02777-19-sf002.tif]

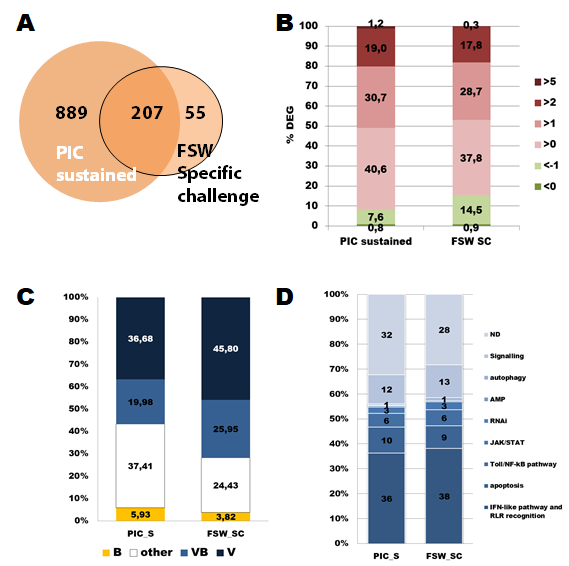

Supplement: FIG S4 [file mBio.02777-19-sf004.tif]

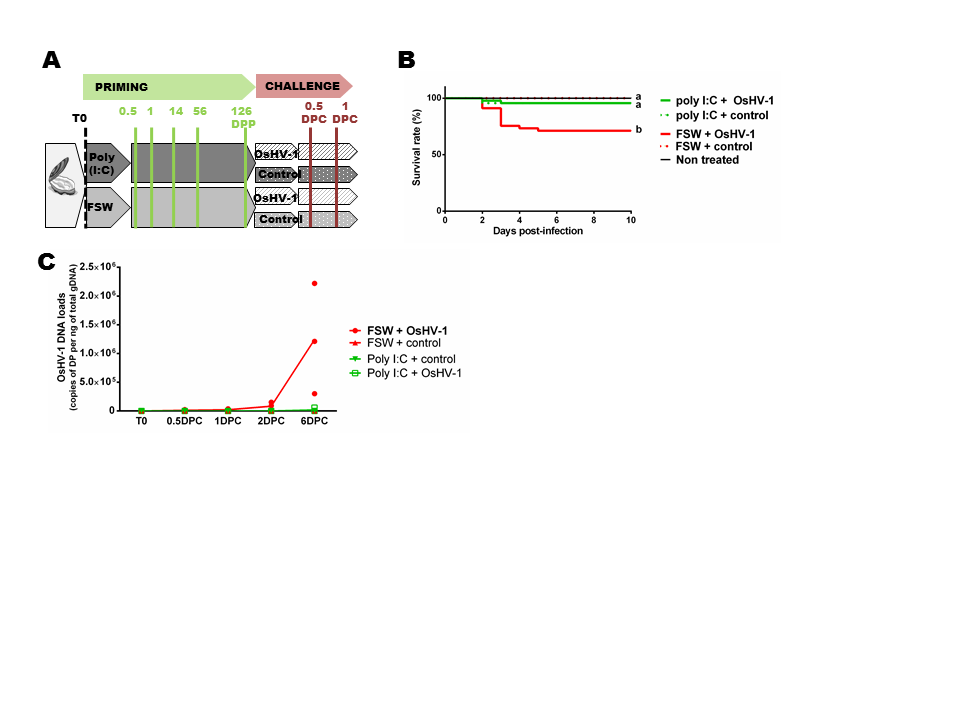

Supplement: FIG S5 [file mBio.02777-19-sf005.tif]
